# Hematologic Complications in Patients Hospitalized with COVID-19 Infection

**DOI:** 10.3390/hematolrep14030031

**Published:** 2022-07-11

**Authors:** Elisa Lin, Ellen Araj, John Markantonis, Hung Luu, Mingyi Chen

**Affiliations:** Department of Pathology, University of Texas Southwestern Medical Center, Dallas, TX 75019, USA; elisa.lin@utsw.edu (E.L.); ellen.araj@utsw.edu (E.A.); john.markantonis@utsw.edu (J.M.); hung.luu@utsw.edu (H.L.)

**Keywords:** COVID-19, hematology, complications, hemolysis

## Abstract

Introduction: This review summarizes data from patients with COVID-19 requiring intensive care unit (ICU) admission. The goals of this study are to showcase some morphological anomalies found in peripheral blood smears from COVID-19 patients and to bring attention to how some hematologic abnormalities in COVID-19 that correspond to disease severity and mortality. Methods: We performed a retrospective analysis of hematologic parameters using peripheral blood smear analysis from 31 COVID-19 patients hospitalized between April 2021 and January 2022. Results: We found abnormal morphology that has not been previously reported. We also report that severe lymphopenia, neutrophilia, acute hemolysis, hematologic malignancies, and increased LDH are associated with ICU admissions, respiratory failure requiring intubation, and poor clinical outcome. Conclusion: We propose these recommendations in the management of COVID-19 patients: 1. Early diagnosis and follow-up of DIC; 2. Optimization of thromboprophylaxis regimen.

## 1. Introduction

Coronavirus disease 2019 (COVID-19) manifests with a wide range of symptoms, from asymptomatic to fatal [1]. Although COVID-19 has been well-studied and characterized, the pathophysiology underlying disease severity and progression remains unclear [1]. Although COVID-19 is primarily a disease of the lower respiratory tract, we now know that it can affect a wide range of systems in the body, including the hematologic system [2]. However, there is conflicting evidence regarding the correlation between the severity of COVID-19 disease and lymphopenia, neutrophilia, and thrombocytopenia; lymphocytosis and neutropenia have been reported in 22% of 486 hospitalized children and 16.7% of pregnant women, respectively, and prevalence of thrombocytopenia varies from 5 to 41.7% in COVID-19 patients [2,3,4,5].

In the hematologic system, it is known that SARS-CoV-2 infects monocytes and endothelial cells, which can result in a cytokine storm-like event and downstream intravascular thrombosis [3]. While arterial and venous thromboses have been reported in patients with COVID-19, DIC is less commonly reported [3,6]. Increased levels of biomarkers such as LDH, D-dimer, ferritin, and C-reactive protein have been studied and have been shown to be associated with poorer outcomes in COVID-19 patients [7,8]. Morphological abnormalities in COVID-19 patients that have been noted include C-shaped, single-lobed neutrophil nuclei, blue-green leukocyte inclusions in circulating neutrophils and/or monocytes, hyperchromatic platelets, and apoptotic and immature granulocytes [7,8].

In this study, we aim to review morphological and hematological abnormalities in patients with COVID-19 requiring intensive care unit (ICU) admission and intubation, and to bring attention to the biomarkers and abnormalities that should lead to change in patient care. We highlight several cases with significant complications impacting the hematopoietic system and hemostasis.

## 2. Materials and Methods

### Study Population

We performed a retrospective analysis of hematologic parameters of 31 hospitalized COVID-19 patients from Clements University Hospital and Parkland Memorial Hospital in Dallas, Texas, between April 2021 and January 2022. Laboratory data, clinical outcomes, demographic data, and past medical history were obtained from electronic medical records. Peripheral blood smears were made by dropping a small amount of the sample and spreading on a glass slide, which was stained with Wright–Giemsa and examined for cells with morphologic abnormalities. All patients had confirmed cases of COVID-19 by nucleic acid amplification tests (NAATs). The study included hospitalized COVID-19 patients with multiple complications and comorbidities, including renal failure, fulminant liver failure, shock, ischemic colitis, bowel ischemia, renal transplant, methicillin-resistant *Staphylococcus aureus* (MRSA) bacteremia, multisystem organ failure, multidrug-resistant (MDR) *Klebsiella*, methicillin-sensitive *Staphylococcus aureus* (MSSA) bacteremia, endocarditis, severe pancreatitis, chronic hypoxemic respiratory failure, lung and liver transplant due to idiopathic pulmonary fibrosis, type 2 diabetes mellitus, essential hypertension, acute kidney injury, morbid obesity, human immunodeficiency virus (HIV), end stage renal disease status post renal transplant, chronic histoplasmosis, cervical cancer, urinary tract infection (UTI), nonischemic cardiomyopathy, allograft dysfunction, secondary hypogammoglobulinemia secondary to immunosuppression, thrombotic thrombocytopenic purpura (TTP) with splenectomy, prostate cancer, cystic fibrosis, sarcoidosis, asthma, sickle cell anemia, pregnancy, and hematologic malignancies (HMs). Severity of disease was assessed by requirement for ICU support, intubation, and outcome (deceased or recovered). IRB approval was attained for this study.

The current study was based on our observations in a small number of cases in a single institution. An ongoing study with multi-center collaboration will provide more insight into the underlying clinico-biological correlation between severe COVID-19 infection and hematological disease.

## 3. Results

### 3.1. Morphological Anomalies in Peripheral Blood Smears from COVID-19 Patients

**Figure 1:**
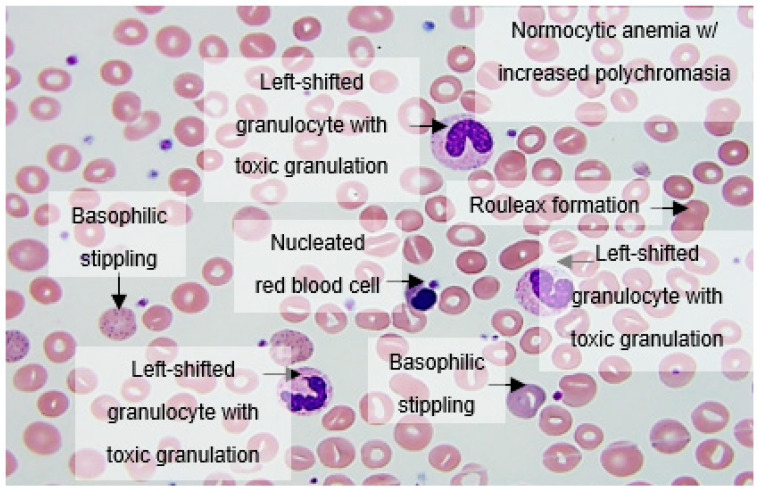
Peripheral blood smear analysis showing increased rouleaux formation, normocytic anemia with increased polychromasia, nucleated red blood cells, basophilic stippling, left-shifted granulocytes with toxic granulations, lymphopenia, and thrombocytopenia.

**Figure 2:**
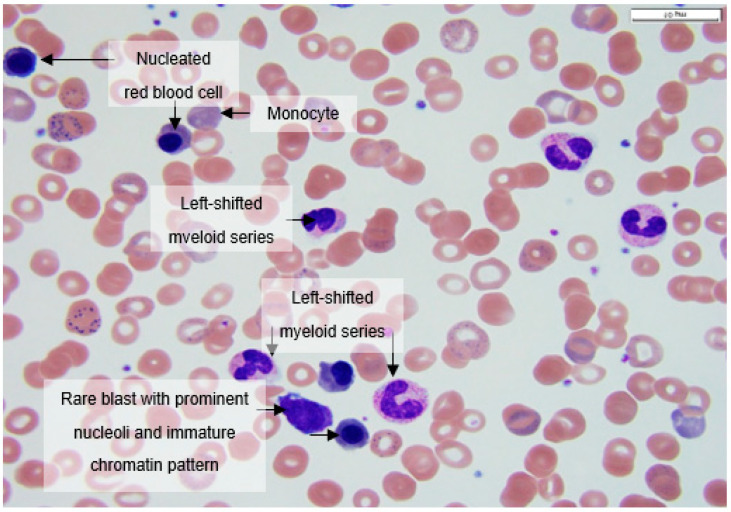
Peripheral smear at 100× magnification showing a nucleated erythroid, a rare blast with prominent nucleoli and immature chromatin pattern, a left-shifted myeloid series with immature promyelocytes and metamyelocytes, and occasional monocytes.

The morphological abnormalities we found included nuclear and cytoplasmic granulations (Figure 1 and Figure 2). In particular, we observed many crowded, dark granulations in the cytoplasm (similar to “toxic” granules) and peripheral light blue agranular areas (Figure 1 and Figure 2). A few cases had many hypogranular cells (Figure 1). The abnormalities in nuclear shape included increased frequency, not only of band forms, but also dysmorphic cells with the total absence of nuclear segmentation, consistent with pseudo-Pelger morphology (Figure 2).

Apoptotic cells were frequently found with either liquefied, granulated nuclear chromatin, or deep blue cytoplasm; they may have been derived from different cell types (i.e., neutrophils and lymphocytes, respectively). Immature granulocytes, especially small myelocytes and metamyelocytes, were also frequently present in early-phase cases, sometimes with small azurophilic granules (Figure 1). Typical features included normocytic anemia with increased polychromasia, circulating nucleated RBCs, basophilic stippling, left-shifted granulocytes with toxic granulations, lymphopenia, and thrombocytopenia (Figure 1).

Peripheral blood smears at 100× magnification often showed nucleated erythroids, rare blasts with prominent nucleoli and immature chromatin pattern, left-shifted myeloid series with immature promyelocytes and metamyelocytes, and occasional monocytes (Figure 2).

### 3.2. Hematologic Laboratory Abnormalities Correlate with Poor Clinical Outcome in COVID-19 Disease

Of the 31 patients in the study, 6 patients were aged 19–30, 18 patients were aged 31–65, and 7 patients were aged 65 or above; 15 patients were Hispanic, 9 were black, 6 were non-Hispanic white, with data unavailable/unknown for 1 patient. Overall, 21 of 31 patients reviewed presented with severe dyspnea and high fever and were admitted to the ICU after being confirmed positive for SARS-CoV-2. Some of the ICU patients (17) developed severe hypoxic respiratory failure and were intubated. On admission, 15 patients (48%) presented with lymphopenia (<1.3 × 10^9^/L) and occasional plasmacytoid reactive lymphocytes; over the course of disease, all 15 experienced worsening lymphopenia. Sixteen patients had absolute neutrophilia (>5 × 10^9^/L) trending downwards a week thereafter (1.48–3.23 × 10^9^/L in seven followed-up cases) and left-shifted granulocytes. Six patients had thrombocytopenia (<150,000/μL), however, three patients had thrombocytosis (>450,000/μL). Further, 14 patients had a positive D-dimer (>0.5 mcg/mL), 9 patients had high fibrinogen (>400 mg/dL), 9 patients had high PT (>14 s) while 3 had low PT (<10 s), 1 patient had INR greater than 3.5, and 7 patients had high PTT (>32.5 s) Eight patients had HMs or were immunosuppressed on admission; five of them required ICU admission and intubation, and ultimately passed away. It is noteworthy that the patients with hematological malignancies such as lymphomas could have had longstanding lymphopenia and elevated LDH prior to COVID-19 disease. Twenty-one patients had elevated LDH on admission (>225 U/L); two had LDH greater than 1000 u/L (Table 1).

## 4. Discussion

### 4.1. Hematologic Abnormalities in COVID-19 Are Related to Disease Progression, Severity, and Mortality

Many cases of severe COVID-19 have been characterized by dysregulated inflammation and coagulation activation [2]. Pro-inflammatory cytokine secretion, microangiopathic vasculopathy, and B-cell secretion of specific SARS-CoV-2 antibodies in infected cells are induced by the SARS-CoV-2 S (spike) protein binding to ACE2 receptors in epithelial cells of the respiratory tract [9]. In particular, COVID-19 disease with marked hematologic abnormalities exhibit a characteristic pattern, similar to a “cytokine storm”, in which inflammatory markers and cytokines dramatically increase and significant lymphopenia develops.

Furthermore, hypercoagulability is common in hospitalized COVID-19 patients [6,10]. Coagulation abnormalities such as prolonged PT and PTT, increased fibrin degradation products, and severe thrombocytopenia can lead to disseminated intravascular coagulation (DIC), which is life-threatening and necessitates continuous vigilance and prompt intervention [10,11,12,13]. Both thrombotic and hemorrhagic pathologies should be noted in these patients, as well as macro- and microthrombosis [13]. Three mechanisms have been proposed to lead to thrombocytopenia in COVID-19 patients. The first is a decrease in platelet production due to myelosuppression either from SARS-CoV-2 or from therapy. The second mechanism is direct platelet destruction by the immune system. The last mechanism purports that platelet aggregration in the lungs results in microthrombi and therefore platelet consumption [4,14]. However, thrombocytosis has also been recorded in patients with COVID-19; it is proposed that some concurrent early-stage bacterial infections may induce neutrophilia, left-shifted granulocytes, and secondary thrombocytosis [15]. Therefore, COVID diagnosis and disease severity must be determined early so extended pharmacological thromboprophylaxis with low-molecular-weight heparin (LMWH) regimen can be started early in the disease course. By doing so, appropriate management can be planned before at-risk patients develop organ failure or shock [2].

### 4.2. Lymphopenia in COVID-19

Lymphopenia in COVID-19 patients has been well documented. In Singapore, approximately 40% of the first 18 hospitalized patients with COVID-19 were found to have lymphopenia [8]. More recently, a report on 69 COVID-19 patients confirmed that approximately 40% of patients had lymphopenia, and 20% had mild thrombocytopenia [8]. Of patients with lymphopenia, 69% showed a reactive lymphocyte population and a lymphoplasmacytoid subset, which was uncommon in the peripheral blood of patients with SARS-CoV-1 (SARS) infections in 2003. No inversion in the CD4+/CD8+ lymphocyte ratio was observed by flow cytometry; however, functional studies suggest that SARS-CoV-2 may impair the function of CD4+ helper and regulatory T-cells and cause rapid exhaustion of cytotoxic CD8+ T-cells [8]. In contrast, several reports have shown only 6% of patients admitted to the hospital for COVID-19 had lymphadenopathies [9]. Immunofluorescence staining and electron microscopy on six autopsies revealed that SARS-CoV-2 directly infects macrophages in lymph nodes (LNs) [9]. Additionally, palpable lymphadenopathies have not been reported in COVID-19 patients [9]. These studies suggest that SARS-CoV-2 belongs to a group of viruses that does not normally induce extensive lymphadenopathies.

There have been several factors identified that may contribute to COVID-19-associated lymphopenia. It has been postulated that since lymphocytes express the ACE2 receptor on their surface, SARS-CoV-2 can directly infect and lyse lymphocytes [10]. It has also been suggested that the substantial cytokine activation often seen in severe COVID-19 disease may be associated with lymphoid organ atrophy, which would impact lymphocyte turnover [10]. Patients who have cancer and therefore are at increased risk for complications from COVID-19 will also have coexisting lactic acidosis, which may also inhibit lymphocyte proliferation [16].

### 4.3. Hematologic Malignancies Put Patients at High Risk of COVID-19 Mortality

Patients with co-existent HMs and COVID-19 are at increased risk of developing severe and life-threatening infections and have a high mortality risk; they have weakened immunity due to cancer as well as treatments they may be receiving, and therefore exhibit reduced response to the COVID-19 vaccines. A case series study found higher fatality rates in COVID-19 patients with multiple myeloma and related diseases compared to COVID-19 patients without multiple myeloma [17]. Studies have shown that patients with lymphoma are particularly vulnerable to SARS-CoV-2 infection, possibly due to the antineoplastic regimens such as BTL or PI3 kinase inhibitors, monocolonal antibodies for CD20, CD30, or CD38, and CAR-T therapy [18,19]. In this study, it should be noted that there is a bias in evaluating biomarkers in patients with hematological malignancies such as lymphomas because the patients may have had longstanding lymphopenia and elevated LDH prior to COVID-19 disease.

Currently, the American Society of Hematology and the International Myeloma Society recommend that high-dose chemotherapy and autologous stem cell transplant should be postponed until the pandemic stabilizes. However, patients with new diagnoses of HMs are especially vulnerable during the pandemic, and therefore may require prompt treatment [16,17,20].

## 5. Conclusions

**Figure 3:**
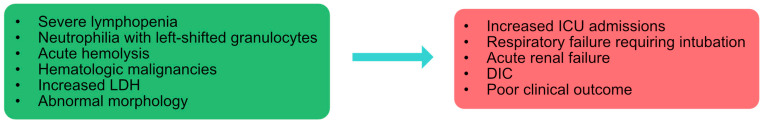
Summary of hematologic complications observed in patients hospitalized with COVID-19 infection.

Based on our clinical observations and a series of studies, we found that severe lymphopenia (<1.3 × 10^9^/L), neutrophilia with left-shifted granulocytes (>5 × 10^9^/L), acute hemolysis, HMs, increased LDH (>225 u/L), and abnormal morphology are usually observed in patients admitted to the ICU who have respiratory failure requiring intubation, acute renal failure, and poor clinical outcome (Figure 3). These findings were consistent with our literature review. This should prompt providers to intervene early to improve outcomes.

In light of these findings, we propose two important additional recommendations in the management of future COVID-19 patients:

1. Early diagnosis and follow-up of DIC by applying the ISTH score, which can determine prognosis and guide appropriate critical care support.
2. Optimization of thromboprophylaxis regimen with LMWH as first-line drug.

## Figures and Tables

**Table 1:** Summary of results from laboratory tests and clinical outcomes.

Manifestation	Number (%)
Severe dyspnea and high fever, admitted to ICU Severe hypoxic respiratory failure requiring intubation	21/31 (70%) 17/21 (81%)
Lymphopenia (<1.3 × 10^9^/L) and occasional plasmacytoid reactive lymphocytes with exacerbation of lymphopenia over course of disease	15/31 (48%)
Absolute neutrophilia (>5 × 10^9^/L) with left-shifted granulocytes Decreasing trend in subsequent week (1.48–3.23 × 10^9^/L)	16/31 (52%) 7/16 (44%)
Hematologic malignancies or immunosuppressionICU admission and intubation, death	8/31 (26%) 5/8 (63%)
Elevated LDH (>225 u/L) LDH > 1000 U/L	21/31 (68%) 2/21 (10%)

## Data Availability

Data available on request from the authors.
